# Comparative Analysis of the Osteogenic Potential of Long-Term Dry-Stored Deciduous and Fresh Permanent Tooth-Derived Dentin Matrix

**DOI:** 10.3390/ma19102147

**Published:** 2026-05-20

**Authors:** Giulia Mazzucchi, Alessia Mariano, Anna Scotto d’Abusco, Alberto De Biase, Marco Lollobrigida

**Affiliations:** 1 Department of Oral and Maxillo Facial Sciences, Sapienza University of Rome, 00161 Rome, Italymarco.lollobrigida@uniroma1.it (M.L.); 2 BeSSA Department of Wellbeing, Health and Environmental Sustainability, Sapienza University of Rome, Rieti Campus, 02100 Rieti, Italy; 3 Department of Biochemical Sciences “Alessandro Rossi Fanelli”, Sapienza University of Rome, 00185 Rome, Italy; anna.scottodabusco@uniroma1.it

**Keywords:** tooth graft, autologous dentin, demineralized dentin matrix, deciduous teeth, bone substitutes, tooth grinder, dentin grinder, bone regeneration

## Abstract

**Highlights:**

**What are the main findings?**

- Long-term dry-stored deciduous teeth retain biologically active dentin matrix.
- Conditioned media from deciduous dentin enhances bone tissue marker gene expression in osteoblasts.
- Osteogenic response confirmed by qRT-PCR, immunofluorescence, and 3D spheroids.

**What are the implications of the main findings?**

- Dry-stored deciduous teeth may represent a viable autologous biomaterial for regenerative applications.
- The results suggest possible advantages over permanent tooth-derived grafts.

**Abstract:**

Autologous tooth-derived grafts are increasingly being investigated for bone regeneration, as dentin shares with bone a mineral phase and an organic matrix rich in type I collagen and non-collagenous proteins. Deciduous teeth are particularly attractive as biomaterials because they are easily obtained after physiological exfoliation, without additional surgical harvesting or donor-site morbidity and may expose a protein-rich matrix after processing. Whether deciduous teeth retain a biologic advantage after prolonged dry storage remains poorly documented. This proof-of-concept ex vivo and in vitro study compared pooled deciduous teeth from six different donors (exfoliated at least 10 years before the experiment and stored dry at room temperature conditions) with six freshly extracted third molars. The teeth were ground using a dedicated device, and conditioned supernatants were collected at 72 h (T1) and 28 days (T2). Osteocalcin, osteonectin, and BMP-2 were quantified by ELISA, and T1 supernatants were applied to human primary osteoblasts to assess the osteogenic response using qRT-PCR and immunofluorescence. Deciduous teeth-conditioned supernatants showed higher osteocalcin and osteonectin release than permanent teeth at both time points, whereas BMP-2 levels were comparable, though with higher values in deciduous samples. In osteoblasts, deciduous teeth-conditioned supernatants induced enhanced osteogenic responses, including greater activation of Collagen I, Osterix, RUNX-2, Osteocalcin, BMP-2 genes, and higher expression of bone-related proteins. Within the limits of this exploratory study, dry-stored deciduous teeth preserved a biologically active dentin matrix and showed a more favorable osteogenic profile than freshly extracted permanent teeth, supporting further investigation into standardized storage protocols and their potential use in regenerative applications.

## 1. Introduction

Successful bone regeneration around teeth and dental implants requires biomaterials capable of providing a mechanically stable scaffold together with biological signals that support cell recruitment, extracellular matrix deposition, and mineralization. Autogenous bone remains the gold standard due to its intrinsic osteogenic, osteoinductive, and osteoconductive properties, yet its clinical use is limited by donor-site morbidity, the need for an additional surgical procedure, restricted availability, and variable resorption over time. These drawbacks have stimulated interest in alternative grafting materials with comparable biological performance but without the associated morbidity. In this context, tooth-derived grafts have gained increasing attention as patient-specific options for oral bone regeneration, supported by growing evidence of their favorable composition, biocompatibility, and regenerative potential [1,2].

Autologous dentin is particularly attractive for the augmentation of atrophic or post-extraction sites, as it exhibits a high affinity with host tissues without eliciting inflammatory reactions and is gradually resorbed and replaced by newly formed bone [3,4]. Its structural similarity to alveolar bone—approximately 70–75% mineral phase and about 20% organic matrix—reflects a composition dominated by hydroxyapatite, type I collagen (≈90%), and a variety of non-collagenous proteins (≈10%), including osteonectin (ON), dentin sialoprotein (DSP), bone sialoprotein (BSP), osteocalcin (OC), and bone morphogenetic proteins (BMPs), all of which contribute to bone healing and mineralized matrix organization [5,6,7,8,9,10]. OC regulates hydroxyapatite crystal formation and matrix mineralization while also functioning as a signaling and adhesion molecule [11]. ON, exhibiting higher concentrations in dentin than enamel, binds to collagen and promotes hydroxyapatite nucleation and crystal growth [5,12]. BMPs, identified within the dentin matrix, further contribute potent osteoinductive activity by driving mesenchymal stem cell differentiation toward the osteoblastic lineage [13,14]. Together, these molecules highlight the biological richness of dentin and provide a molecular basis for its osteoconductive and osteoinductive potential.

Processing parameters further modulate the regenerative behavior of dentin-derived grafts. Demineralization enhances the release of BMP-2 and other signaling molecules otherwise sequestered in the mineral phase, thereby increasing the osteoinductive potential of the matrix [15]. Particle size and the degree of demineralization influence cellular attachment, resorption dynamics, and new bone formation, with partially demineralized larger particles showing particularly favorable in vivo outcomes [16].

Given these attributes, extracted teeth have been repurposed as autologous grafting materials in regenerative dentistry [17]. Among tooth-derived biomaterials, deciduous teeth (DT) are of special interest: they become available physiologically, require no additional harvesting procedure, and may offer physicochemical advantages such as a higher surface area and faster exposure of the organic matrix after processing [18]. In DT, demineralization preserves type I collagen and increases BMP-2 bioavailability, supporting in vitro osteo-differentiation [19], while clinical and histological evidence suggests that DT-derived material can be grafted into alveolar defects and support implant placement [20].

The biological rationale for testing DT is reinforced by developmental studies showing that odontoblasts produce OC and ON, with distinct extracellular distributions also in deciduous dentition [5]. More recently, Mazzucchi et al. demonstrated that ground extracted teeth release BMP-2, OC, and ON over time, indicating that tooth-derived particulate is not merely a passive scaffold but a source of bioactive osteogenic signals [21]. However, a direct comparison between long-term dry-stored DT and freshly extracted permanent teeth (PT) is still lacking, particularly regarding soluble factor release and downstream osteoblast behavior.

The rationale for evaluating dry-stored DT lies in their practical availability as a ready-to-use autologous resource for adult patients who have preserved them. Accordingly, this exploratory proof-of-concept study does not aim to establish an absolute biological comparison between DT and PT, but rather to assess the clinical viability of stored dental tissues as an alternative to autologous bone. Specifically, we compared the presence and residual biological activity of key osteogenic proteins between DT and freshly extracted third molars.

## 2. Materials and Methods

### 2.1. Study Design and Sample Source

The study was conceived as a comparative ex vivo/in vitro investigation of the soluble protein release from processed tooth-derived particulate and of the biologic response induced in human primary osteoblasts (hOBs).

Two groups were compared: (i) DT from six independent donors (DT group) and (ii) six fresh permanent third molars (PT group). To ensure sufficient material for the grinding procedure and subsequent experiments, multiple deciduous teeth from each individual donor were pooled into a single experimental unit, resulting in six distinct DT samples (Table 1). The choice of long-term (16–32 years) dry-stored deciduous teeth was intentionally made to reflect the most common home-storage modality. Storage conditions included the use of closed containers (not sealed) at room temperature (approximately 23–28 °C).

For deciduous tooth donors, inclusion criteria were: age ≥ 22 years; deciduous teeth stored in a clean and dry environment. Exclusion criteria included: insufficient number of teeth to obtain at least 0.5 g of particulate; non-dry storage conditions; teeth severely compromised by caries or structural defects; and systemic diseases or syndromes known to affect dental mineralization. For permanent tooth donors, inclusion criteria were: age between 20 and 35 years; clinical indication for extraction of maxillary third molars; intact teeth not subjected to odontotomy; good general health. Exclusion criteria included: teeth severely compromised by caries; and systemic diseases or syndromes capable of impairing dental mineralization (Table 2).

### 2.2. Sample Preparation and Smart Dentin Grinder Procedure

Following extraction, PT were cleaned of enamel and soft tissues using a tungsten carbide bur, then dried and stored in a sterile environment. For the deciduous group, multiple teeth belonging to the same donor were placed into the sterile grinding chamber and processed as a single experimental unit. The pooling was performed strictly on a per-donor basis, ensuring that no cross-contamination or mixing occurred between samples from different individuals.

Both PT and DT samples were processed using a dedicated device (Smart Dentin Grinder™, KometaBio Inc., Cresskill, NJ, USA), involving a 3-s grinding phase followed by a 10-s sorting cycle. This mechanical procedure distributed the resulting particulate into two distinct drawers: an upper compartment contained granules ranging from 300 to 1200 μm in diameter (porosity of 2–14 μm) and a lower compartment collected fine particles smaller than 300 μm. For all subsequent experimental phases, only the 300–1200 μm fraction was utilized, while the particles smaller than 300 μm were discarded, consistent with clinical standards to prevent an excessive inflammatory response, which is often triggered by fine particles [22,23].

**Figure 1:**
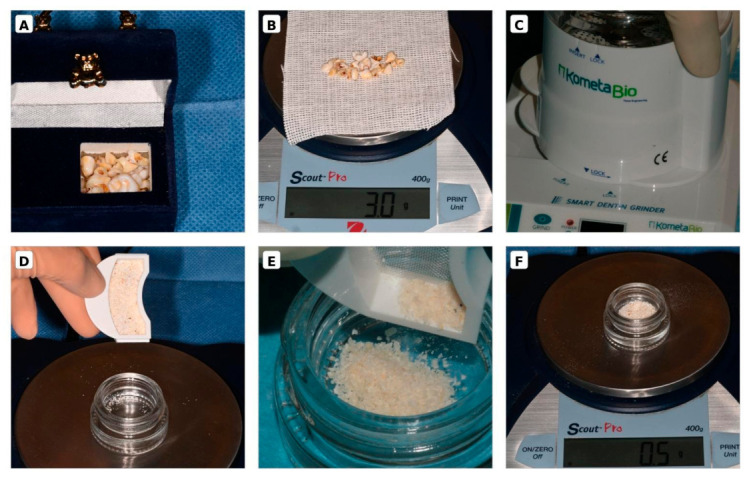
Procedural workflow for autologous dentin graft preparation. (**A**) Retrieved deciduous teeth stored by the patient in a storage container. (**B**) Teeth cleaned, dried, and weighed (3.0 g) on a digital scale (Scout Pro, 400 g capacity) before grinding. (**C**) Grinder device with the grinding chamber in place. (**D**) Particulate collected from the upper drawer compartment (particle size range 300–1200 µm according to the manufacturer). (**E**) Close-up view of the dentin granules being transferred into a sterile glass dappen dish for subsequent chemical processing. (**F**) Calibrated amount of graft particulate (0.5 g) weighed to standardize the decontamination and storage steps.

**Figure 2:**
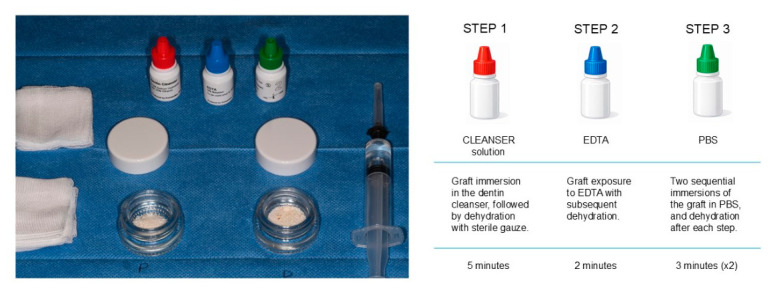
Standardized cleaning workflow applied to the particulate collected from the upper compartment (see Figure 1D). Step 1: immersion of the graft in a NaOH/ethanol cleanser solution for 5 min. Step 2: conditioning with EDTA for 2 min to modify the graft surface. Step 3: neutralization through two consecutive washes with phosphate-buffered saline (PBS).

For standardization, 0.5 g of granules from each sample were processed according to the manufacturer’s protocol. The particulate was decontaminated in 0.5 M NaOH and 20% ethanol for 5 min, dehydrated, and then conditioned with EDTA for 2 min. After a second dehydration, the material underwent two 3-min PBS washes to neutralize the pH. The resulting granules were stored in 2 mL of PBS at 4 °C (Figure 1 and Figure 2).

The experimental workflow was aimed at evaluating the soluble protein release from the processed material and the biologic activity of the corresponding conditioned supernatants. Supernatants were harvested at 72 h (T1) and 28 days (T2) for the biochemical assays. The T1 supernatants were subsequently diluted 1:3 in cell culture medium and used for osteoblast stimulation.

### 2.3. ELISA

The amounts of OC, ON, and bone morphogenetic protein 2 (BMP-2) in both the permanent and DT-conditioned supernatants collected at 72 h (T1) and 28 days (T2) were determined using enzyme-linked immunosorbent assay kits (Fine Test ELISA, Fine Biotech Co., Ltd., Wuhan, China) according to the manufacturer’s instructions. Optical density absorbance was measured at 450 nm by a microplate reader (NeoBiotech, Holden, MA, USA).

### 2.4. Human Primary Osteoblasts: Culture and Analysis

#### 2.4.1. Cell Culture

Human primary osteoblasts (hOBs), obtained from patients undergoing arthroplasty surgery were isolated from femoral and tibial condyles, as previously described [24]. Full ethical consent was obtained from all donors. The Research Ethics Committee, ASL Lazio 2 (0047966) approved the tissue explantations. Briefly, the bone fragments were washed in sterile PBS, minced, and treated with 1 mg/mL collagenase type IV plus 0.25% trypsin solution for 1 h at 37 °C under gentle agitation. After isolation, hOBs were grown to 80% confluence in McCoy’s medium (Sigma Aldrich, Co., Ltd., Saint Louis, MO, USA), supplemented with L-glutamine, penicillin/streptomycin, Fungizone, and 15% fetal bovine serum (FBS) (all purchased from Sigma Aldrich). The experiments were performed with cells at first passage (P1).

#### 2.4.2. Cell Treatment

Cells were plated in cell devices, cultured to 80% confluence, and then treated with permanent or DT-conditioned supernatant collected at T1, with 1:3 dilution in cell medium, for the required times. Untreated cells served as controls (CTL).

#### 2.4.3. RNA Extraction and qRT-PCR

Total RNA from the treated and untreated cells was extracted with a Blood/Tissues Total RNA extraction kit (Fisher Molecular Biology, Trevose, PA, USA). Reverse transcription was performed according to the manufacturer’s instructions using Meridian Bioscience Reverse Transcriptase (Bioline reagent Ltd., London, UK). Quantitative real-time PCR (qRT-PCR) analysis was performed on an ABI Prism 7300 platform (Applied Biosystems, Thermo Fisher Scientific, Waltham, MA, USA). Amplification was carried out using SensimixPlus SYBR Master Mix (Bioline). Primers, synthesized by Bio-Fab Research and designed using Primer Express software v1.4.0 (Applied Biosystems), are reported in Table 3. Data were analyzed by the 2^−ΔΔCt^ method, determining transcript abundance relative to the 18S rRNA housekeeping gene.

#### 2.4.4. Spheroids Preparation

Three-dimensional (3D) spheroid cultures were prepared using the Sphericalplate 5D system (Kuglermeiers, Erlenbach, Switzerland). Briefly, 5 × 10^4^ cells were plated in the wells, left untreated (CTL) or treated with permanent or DT-conditioned supernatants collected at T1 with 1:3 dilution in cell medium, for 7 days. Cells were then fixed and prepared for the immunofluorescence assay. Each experiment was repeated at least three times independently.

#### 2.4.5. Immunofluorescence and Densitometric Analysis

Proteins were visualized by immunofluorescence. Cells plated at a density of 8 × 10^3^/cm^2^ were left untreated (CTL) or treated with permanent or DT-conditioned supernatant collected at T1 with 1:3 dilution in cell medium. After treatment, both the seeded cells and spheroids were washed in phosphate-buffered saline (PBS), fixed in ethanol for 15 min, and permeabilized with 0.5% Triton-X 100 in PBS for 10 min at room temperature (RT). Cells were then blocked in 3% bovine serum albumin in PBS for 30 min at RT and incubated for 1 h at RT with anti-Runt-related transcription factor 2 (RUNX-2) rabbit monoclonal primary antibody (bs-1134R, BIOS Antibodies, Woburn, MA, USA) diluted 1:300, anti-Osterix (OSX) mouse monoclonal antibody (H00121340-M01, AbNova, Taipei City, Taiwan) diluted 1:50, anti-Collagen I mouse monoclonal primary antibody (sc-8786, Santa Cruz Biotechnology, Dallas, TX, USA) diluted 1:100, and anti-Bone Sialoprotein (BSP) rabbit monoclonal primary antibody (ab-81521, Immunological Sciences, Rome, Italy) diluted 1:100. After PBS washing, cells were incubated for 1 h at RT with Alexa Fluor 595 donkey anti-mouse, Alexa Fluor 488 goat anti-mouse, Alexa Fluor 595 donkey anti-rabbit, or Alexa Fluor 488 goat anti-rabbit secondary antibodies diluted 1:400 (Invitrogen, Thermo Fisher Scientific, Waltham, MA, USA). After washing, nuclei were counterstained with DAPI (Invitrogen, Thermo Fisher Scientific). Images were captured using a Leica DM IL LED optical microscope with an AF6000 modular microscope system (Leica Microsystems, Milan, Italy). Densitometric quantification was performed for RUNX-2, osterix (OSX), Collagen I, and bone sialoprotein (BSP) with ImageJ (version 1.54p, https://imagej.nih.gov/ij/, accessed on 10 April 2026) using the integrated density values obtained from the immunofluorescence experiments.

### 2.5. Statistical Analysis

For ELISA assays, statistical analysis was performed using a nonparametric one-way ANOVA (Kruskal–Wallis test) and post hoc Mann–Whitney U testing adjusted by Bonferroni correction for multiple tests in order to compare the groups at each time point. Data were plotted in histograms using Prism 5.0 software (GraphPad Software, San Diego, CA, USA). Regarding the experiments on hOBs, all data were obtained from at least three independent experiments, each performed either in duplicate or triplicate. Data were statistically analyzed with two-way repeated-measures ANOVA followed by Bonferroni’s multiple comparison test, using Prism 5.0 software (GraphPad Software). A *p* value < 0.05 was considered significant.

## 3. Results

### 3.1. Soluble Release Profile of Deciduous and Permanent Tooth-Derived Samples

**Figure 3:**
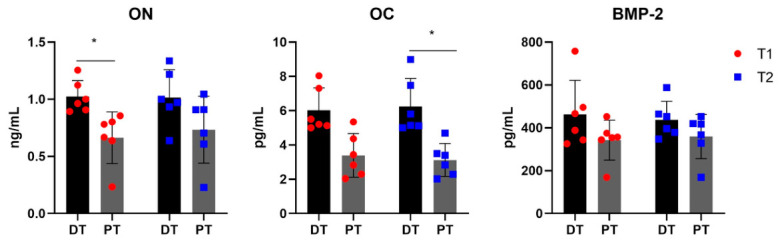
ELISA quantification of osteonectin (ON), osteocalcin (OC), and BMP-2 in conditioned supernatants obtained from deciduous teeth (DT; black bars) and permanent teeth (PT; gray bars) at T1 (72 h; individual values are represented as red dots) and T2 (28 days; individual values are represented as blue squares). The results are reported as ng/mL for ON and pg/mL for OC and BMP-2 and are expressed as mean ± standard deviation (SD). * *p* < 0.05 PT vs. DT.

The ELISA analysis of tooth-conditioned supernatants disclosed a more favorable soluble protein profile for the deciduous group than for the permanent group, although the statistical pattern differed according to the marker considered (Figure 3). ON was consistently higher in deciduous samples at both collection times, and the statistical analysis indicates a significant difference between DT and PT at T1 (*p* = 0.0481 for T1 PT vs. DT; *p* > 0.1 for T2 PT vs. DT). This early enrichment in ON is relevant because it is implicated in collagen interaction and extracellular matrix organization. OC showed the same directional trend, with higher mean values in deciduous samples at both T1 and T2. However, the statistical significance was displayed only at T2 (*p* = 0.0540 for T1 PT vs. DT and *p* = 0.0140 for T2 PT vs. DT). Since OC is associated with osteoblast maturation and matrix mineralization, this finding suggests that the deciduous material retained a protein cargo with particular relevance to the later phases of matrix maturation. BMP-2 was detectable in both groups at both time points. The mean values remained higher in the deciduous samples, but no statistical significance was found (*p* > 0.5 for both T1 and T2 PT vs. DT). Taken together, these data indicate that the main differences between the two groups concerned ON and OC rather than BMP-2, while still confirming that both tooth-derived materials released measurable osteogenic mediators.

### 3.2. hOB Transcriptional Response to Tooth-Derived Supernatants

**Figure 4:**
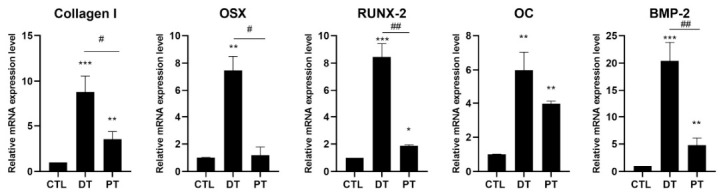
Relative mRNA expression of Collagen I, Osterix (OSX), Runt-related transcription factor 2 (RUNX-2), osteocalcin (OC), and bone morphogenetic protein-2 (BMP-2) in human primary oste-oblasts treated with T1 supernatants from deciduous teeth (DT) or permanent teeth (PT) versus untreated cells (CTL). mRNA was analyzed by qRT-PCR. Collagen I, OSX, RUNX-2, OC, and BMP-2 mRNA levels were reported as relative mRNA expression levels with respect to 18S rRNA (2^−∆∆Ct^ method). Results are expressed as mean ± standard deviation (SD) of data obtained by three independent experiments. ** *p* < 0.01, *** *p* < 0.005 DT-treated cells vs. CTL and * *p* < 0.05 and ** *p* < 0.01 PT treated cells vs. CTL. # *p* < 0.05 and ## *p* < 0.01 PT treated cells vs. DT-treated cells.

The transcriptional analysis of the hOBs treated with T1 supernatants from DT and PT showed that both tooth-derived conditions activated osteogenic genes compared to untreated cells, CTL, but the deciduous condition consistently induced a higher response (Figure 4). The highest relative increase was observed for BMP-2, followed by Collagen I, Runt-related transcription factor 2 (RUNX-2), Osterix (OSX), and finally OC in DT group. The PT supernatant was not biologically inert, because all five targets remained above control and reached statistical significance. However, the magnitude of induction was lower than in the deciduous group.

### 3.3. hOB Protein Factors Response to Tooth-Derived Supernatants

**Figure 5:**
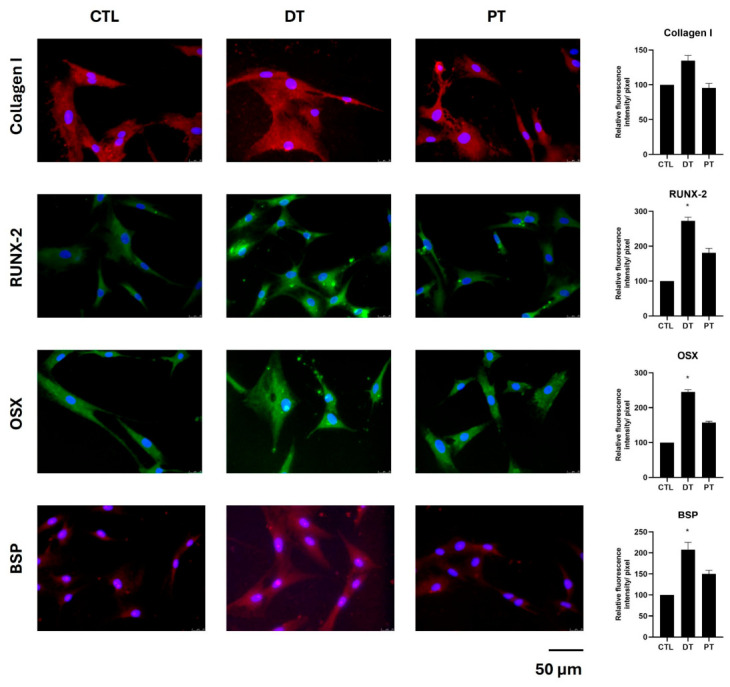
Representative immunofluorescence images and related densitometric analyses of Collagen I (in red), RUNX-2 (in green), OSX (in green), and Bone Sialoprotein (BSP) (in red) in hOB monolayers left untreated (CTL) or after treatment with deciduous tooth (DT) or permanent tooth (PT) T1 supernatants. Nuclei were counterstained with DAPI (blue) (original magnification 40×). Scale bar: 50 µm. The histogram represents the pixel intensities in the region of interest, obtained by ImageJ (version 1.54p). Results are expressed as mean ± standard deviation (SD) of data obtained by three independent experiments. * *p* < 0.05, DT-treated cells vs. CTL.

The protein expression analyses corroborated the gene-expression results (Figure 5 and Figure S1). The levels of Collagen I, RUNX-2, OSX, and Bone Sialoprotein (BSP) were visibly higher in the DT group compared to the PT group. The differences between groups were most convincing for RUNX-2, OSX, and BSP, for which the deciduous group showed significant increase compared to CTL. Collagen I followed the same directional trend, with a higher expression in DT than PT. Biologically, this pattern is coherent with an early activation of osteoblastic commitment (RUNX-2 and OSX) followed by the enhancement of the matrix-related phenotype (Collagen I and BSP).

### 3.4. Collagen I Expression in 3D hOB Spheroids

**Figure 6:**
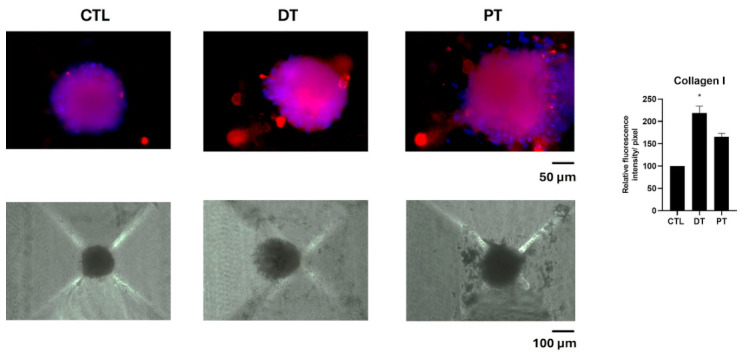
Collagen I (in red) expression in 3D hOB spheroids left untreated (CTL) or after treatment with deciduous tooth (DT) or permanent tooth (PT) T1 supernatants. The upper row shows immunofluorescence images (original magnification 20×) and scale bar: 50 µm, the lower row bright-field images (original magnification 10×) and scale bar: 100 µm. The graph reports the related densitometric analysis. Nuclei were counterstained with DAPI (blue). The histogram represents the pixel intensities in the region of interest, obtained by ImageJ. Results are expressed as mean ± standard deviation (SD) of data obtained by three independent experiments. * *p* < 0.05, DT-treated cells vs. CTL.

The 3D spheroid model yielded a Collagen I distribution consistent with the monolayer data but in a more physiological culture setting (Figure 6). DT-conditioned supernatant induced a higher statistically significant Collagen I expression than PT-conditioned supernatant. The corresponding bright-field images document spheroid morphology under the same experimental conditions and support the interpretation that the deciduous-conditioned medium promoted a more collagen-rich extracellular environment. CTL spheroids showed the smallest, with a mean diameter of 145 μm ± 10 μm, and most compact structures, with more regular borders. DT-treated spheroids exhibited intermediate dimensions (with a mean diameter of 205 μm ± 15 μm) while maintaining a compact morphology, whereas PT spheroids appeared larger (with a mean diameter of 215 μm ± 25 μm) and less compact, with more irregular and jagged edges, reflected by the higher measured SD value.

Because 3D systems provide a setting closer to spatial matrix assembly than conventional monolayers, this result strengthens the overall evidence that deciduous tooth-derived soluble factors exert a more favorable effect on osteoblast behavior.

## 4. Discussion

The growing interest in tooth-derived grafts reflects a broader shift toward autologous materials capable of supporting bone regeneration without the drawbacks associated with traditional grafts (bone autograft, allograft, and xenograft). Freshly processed dentin has already demonstrated favorable osteoconductive and osteoinductive properties, positioning it as a promising alternative to autogenous bone in those clinical scenarios [25,26]. However, a largely unexplored question concerns whether teeth stored for long periods outside controlled environments, such as the deciduous teeth that many individuals keep for personal reasons, might still retain a biologically meaningful protein reservoir. The present study was designed to address this gap, evaluating not only the measurable release of dentin-derived proteins but also their functional impact on human osteoblasts. Surprisingly, despite more than one decade of dry domestic storage, deciduous teeth exhibited a robust and quantifiable biological activity, prompting a reconsideration of how durable the dentinal matrix may be over time.

This persistence of biological potential after more than ten years raises questions about the stability of the dentinal organic matrix. Unlike ex vivo soft tissues, where proteins undergo rapid denaturation and proteolysis once removed from their physiological environment, the mineral component of dentin acts as a physical barrier. Though advanced biochemical analyses such as Amino Acid Racemization or Mass Spectrometry were not performed to quantify protein degradation kinetics, the induction of a complete osteogenic program in human primary osteoblasts served as a definitive functional readout. The mineral component of the dentin likely acted as a biochemical shield, preserving the structural folding and bioactivity of essential growth factors for over a decade. This “mineral shield” effect is well-documented in paleoproteomic research, where dental and bone collagen as well as other non-collagenous proteins have been successfully sequenced from samples dating back thousands of years, demonstrating the extraordinary capacity of the hydroxyapatite lattice to stabilize organic molecules against degradation [27,28]. However, at the time of clinical use, this mineral shield must be partially removed to maximize the bioavailability of the entrapped signaling molecules. Current clinical trends thus include an EDTA conditioning step to bypass the mineral barrier, thereby exposing the collagen-bound proteins to the surrounding environment. In line with this concept, our ELISA assays confirmed that long-term stored, EDTA-conditioned DT were still able to release measurable amounts of dentin-derived proteins into solution, including ON, OC, and BMP-2 at both T1 and T2. Notably, the concentrations observed in deciduous samples were higher than those released by freshly extracted permanent teeth.

Regarding release profiles, our results suggest a differential preservation pattern among dentinal proteins. Whereas ON and OC displayed statistically significant increases in the deciduous group, BMP-2 followed the same directional trend but remained below the threshold of statistical significance. This may depend on the small sample size but might also reflect a higher susceptibility of larger growth factors to long-term degradation. The discrepancy between the non-significant difference in exogenous BMP-2 levels (ELISA) and the significant upregulation of endogenous BMP-2 mRNA (qRT-PCR) suggests the hypothesis of an amplification mechanism, likely driven by a positive feedback loop: the initial contact with dentin-derived growth factors triggers RUNX-2 activation, which in turn enhances the cells’ own BMP-2 transcription as previously described in the literature [29]. Alternatively, it is possible that the osteoblastic response observed in our study was driven by a synergistic effect of the entire dentin matrix signature rather than the activity of a single growth factor. Nevertheless, the fact that we observed not merely the presence of a single molecule, but a general residual biological activity, evidenced by the significant upregulation of BMP-2, Collagen I, RUNX-2, OSX, and OC genes in osteoblasts, confirms that three-dimensional proteins’ conformation has been preserved, allowing them to effectively trigger the osteogenic cascade. Of particular interest were the results regarding RUNX-2 and OSX, two transcription factors central to osteoblast lineage commitment and progression toward a mature osteoblastic phenotype. The concurrent upregulation of Collagen I, OC, and endogenous BMP-2 supports the interpretation that the DT-conditioned supernatant triggered a broader osteogenic program rather than an isolated change in a single marker, as also supported by immunofluorescence findings.

The biologic performance observed for the DT-derived dentin matrix represents a particularly intriguing and unreported finding that, to the best of our knowledge, has not been described in the literature and therefore warrants further investigation. We can hypothetically link this phenomenon to the distinct chronobiology of tooth development. Deciduous dentin is formed during fetal life and early infancy, periods characterized by peak systemic levels of growth hormone (GH) and insulin-like growth factor 1 (IGF-1) [30,31], which are known to stimulate odontoblast activity and enhance the secretion of bone-related matrix proteins within dentin [32,33]. While this represents a chronological confounder, it is also the likely driver of the observed differences, as the matrix composition reflects the specific biochemical milieu present during its formation. In addition, the odontogenesis of primary teeth proceeds more rapidly than that of permanent teeth, resulting in a lower mineralization and consequently a higher organic-to-inorganic ratio [34]. A parallel can be drawn with bone physiology: bones in children are characterized by higher elasticity and collagen content, whereas adult bone becomes more mineralized and brittle [35]. This biological affinity between bone and dentin explains why DT might retain a more dynamic and protein-rich matrix, making them a valuable reservoir for regenerative signaling molecules.

The long dry storage represents another highly original aspect of this study. This storage modality allowed us to evaluate the biological viability of dentin matrix in a worst-case scenario regarding protein preservation, which is critical for assessing the real-world feasibility of autologous grafting from stored primary teeth. Although domestic storage cannot be considered a standardized and professional banking method, the results suggest that the hard-tissue matrix may preserve biologically relevant proteins for long periods even in uncontrolled but clean conditions. This observation is coherent with previous work showing the conservation of growth factors and extracellular matrix proteins in ancient human bone and tooth tissues [36]. These findings open significant perspectives for clinical practice. If simple, clean domestic storage is sufficient to preserve the biological properties of dentin over long periods, the need for dedicated tooth banks may not be essential for this purpose. While these facilities would remain valuable for the banking of dental pulp stem cells, the storage of dentin itself might not require specialized infrastructure. This would make autologous regenerative materials more readily accessible, simplifying the workflow for clinicians and patients.

These preliminary findings indicate that long-term stored DT may retain a surprisingly robust biological profile, capable of releasing a mixture of osteogenic proteins and of activating multiple levels of osteoblastic metabolism. The convergence of biochemical, transcriptional, and 3D culture data supports the interpretation that the deciduous dentin matrix remains functionally competent despite decades of non-standardized storage. Rather than reflecting the action of a single factor, the biologic performance of DT appears to arise from a composite matrix signature rich in structural proteins, non-collagenous components, and residual signaling molecules that collectively promote a more favorable osteogenic environment than freshly processed permanent teeth. This integrated evidence reinforces the biological plausibility and the clinical potential of DT-derived grafts as a viable autologous resource for bone regeneration.

This study has some limitations. The sample size was limited, and the two tooth groups differed not only by anatomical type but also by storage history, introducing a degree of heterogeneity. However, it is important to note that long-term dry storage is a condition that typically leads to protein degradation and a loss of bioactivity. The fact that, despite this suboptimal long-term storage, the DT-derived matrix still exhibited a significant release of proteins and osteogenic potential suggests that the intrinsic biological advantages of deciduous dentin are robust enough to compensate for any time-dependent decay. Therefore, this limitation provides *a fortiori* evidence. Moreover, only the soluble fraction released into PBS was analyzed, and the in vitro assays were performed with conditioned media rather than direct cell–particle interaction, which would more closely mimic the clinical scenario. Finally, because the most pronounced differences involved ON and OC while BMP-2 remained within a comparable range, the biologic activity of deciduous samples likely reflects a composite matrix signature rather than a single mediator. Despite the small sample size, the consistent trends observed in this study provide a strong proof-of-concept for the long-term stability of dentin bioactivity. Future studies should include larger samples and a broader physicochemical and proteomic characterization to better define protein degradation kinetics. Furthermore, a comparison between different storage modalities (e.g., fresh vs. dry-stored vs. cryopreserved) would be beneficial to optimize the osteogenic potential, while in vivo models remain necessary to assess the true osteoconductive and osteoinductive effect of this matrix.

## 5. Conclusions

Within the limits of this exploratory ex vivo/in vitro study, long-term stored deciduous teeth retained a biologically active dentin matrix capable of eliciting a stronger osteogenic response than fresh permanent teeth. The overall profile emerging from our analyses suggests that deciduous tooth-derived material may offer a favorable combination of matrix components and osteoinductive cues, supporting its potential as a graft material for bone regeneration procedures. Further validation through in vivo studies, standardized processing workflows, and dedicated clinical trials will be essential to determine its translational relevance.

## Figures and Tables

**Table 1:** Overview of the experimental design used for the comparative analysis of deciduous and permanent tooth-derived conditioned media.

Experimental Arm	Material Source	Time Points/Treatment	Readouts
Deciduous teeth	n = 6; extracted/exfoliated >10 years earlier; dry-stored	Conditioned supernatant collected at T1 (72 h) and T2 (28 days)	ELISA for OC, ON, BMP-2; T1 used for hOB treatment
Permanent teeth	n = 6; freshly extracted maxillary third molars	Conditioned supernatant collected at T1 (72 h) and T2 (28 days)	ELISA for OC, ON, BMP-2; T1 used for hOB treatment
Osteoblast assays	Human primary osteoblasts (P1)	T1 supernatant diluted 1:3 in cell medium; untreated cells used as CTL	qRT-PCR and immunofluorescence

**Table 2:** Summary of donor demographics.

	Deciduous Teeth Donors						Permanent Teeth Donors					
Donor ID	1	2	3	4	5	6	1	2	3	4	5	6
Age	32	24	24	22	38	22	32	22	20	20	26	23
Sex	F	F	F	F	F	M	F	F	M	M	F	M

**Table 3:** Primer sequences used for qRT-PCR analysis. The Accession Numbers are indicated.

Gene	Primer Sequences (Fw-Rv)
*RUNX-2* NM_001024630.4	5′-GCAAGGTTCAACGATCTCAGATT-3′ 5′-GTGAAGACGGTTATGTGCAAGGT-3′
*Collagen I* NM_000088	5′-AAGGGTGAGACAGGCGAACA-3′ 5′-GACCCTGGAGGCCAGAGAA-3′
*OSX* NM_001173467.3	5′-AGAGCAACTGCTGGAGATC-3′ 5′-AAGCAGTGGTCTAGAGAGCC-3′
*OC* NM_199173	5′-AGCAAAGGTGCAGCCTTTGT-3′ 5′-GCGCCTGGGTCTCTTCACT-3′
*BMP-2* NM_001200	5′-CCAGCTGTAAGAGACACCCTTTG-3′ 5′-AGCCACAATCCAGTCATTCCA-3′
*18S* NM_003286	5′-CGCCGCTAGAGGTGAAATTC-3′ 5′-CATTCTTGGCAAATGCTTTCG-3′

## Data Availability

The raw data supporting the conclusions of this article will be made available by the authors on request.

## Supplementary Materials

The following supporting information can be downloaded at: https://www.mdpi.com/article/10.3390/ma19102147/s1, Figure S1: Representative immunofluorescence images and related densitometric analyses of Collagen I, RUNX-2, OSX, and Bone Sialoprotein (BSP) in hOB monolayers left untreated (CTL) or after treatment with deciduous tooth (DT) or permanent tooth (PT) T1 supernatants.

## Abbreviations

The following abbreviations are used in this manuscript:

BMP-2	Bone Morphogenetic Protein-2
BSP	Bone Sialoprotein
CTL	Control
DT	Deciduous Teeth
EDTA	Ethylenediaminetetraacetic Acid
ELISA	Enzyme-Linked Immunosorbent Assay
FBS	Fetal Bovine Serum
hOBs	Human Primary Osteoblasts
OC	Osteocalcin
ON	Osteonectin
OSX	Osterix
PBS	Phosphate-Buffered Saline
PT	Permanent Teeth
qRT-PCR	Quantitative Real-Time Polymerase Chain Reaction
RUNX-2	Runt-Related Transcription Factor 2
SD	Standard Deviation
T1	Time Point 1 (72 h)
T2	Time Point 2 (28 days)
3D	Three-Dimensional
