# Supplementary material for: Comparative Analysis of the Osteogenic Potential of Long-Term Dry-Stored Deciduous and Fresh Permanent Tooth-Derived Dentin Matrix
Source: Materials (Basel). 2026 May 20;19(10):2147. doi: 10.3390/ma19102147 (PMC13208260; doi:10.3390/ma19102147)
Supplement: Supplementary file 1 [file materials-19-02147-s001.zip › materials-4298710-supplementary.pdf]

SUPPLEMENTARY MATERIALS

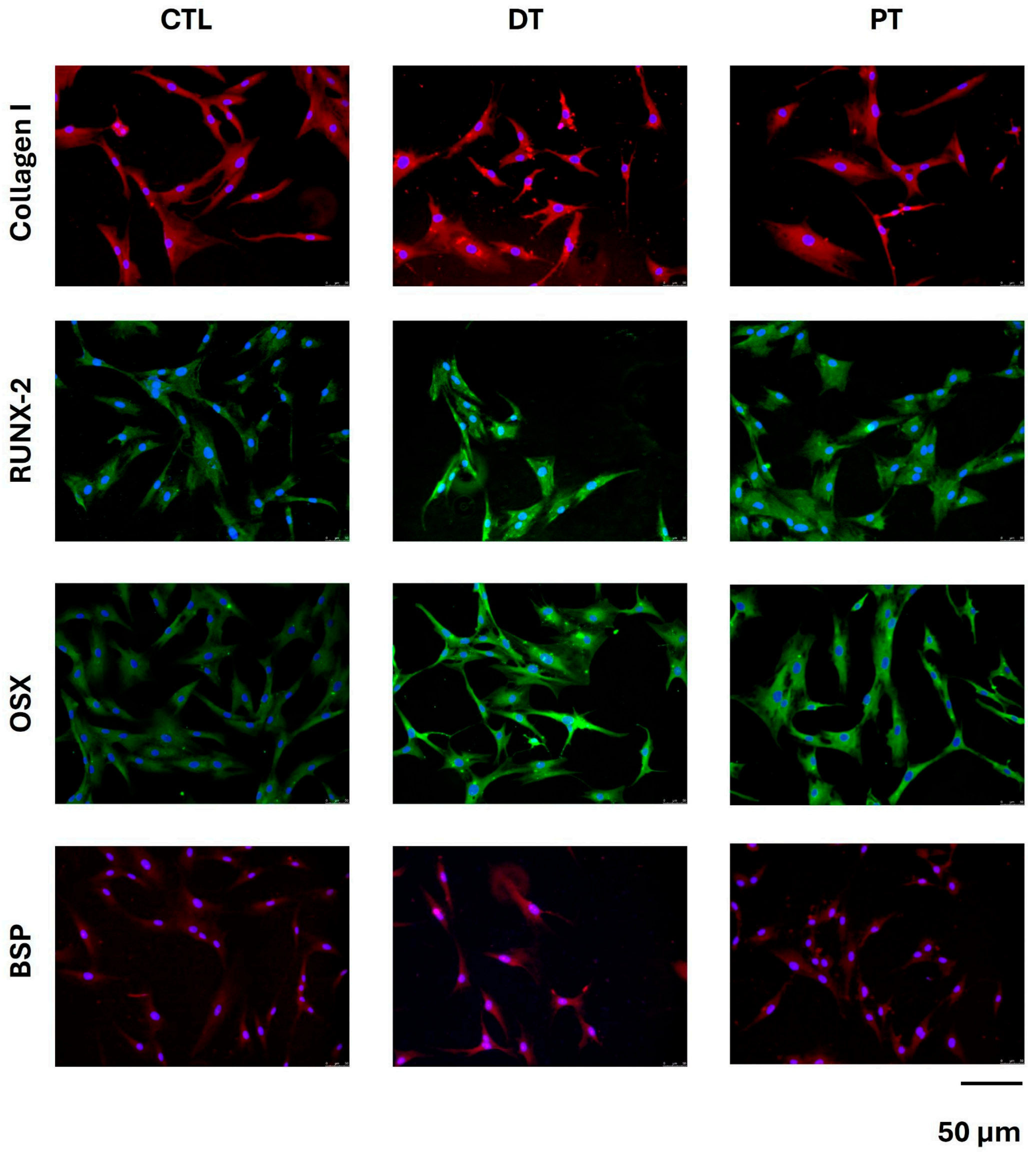

Figure S1. Representative immunofluorescence images and related densitometric analyses of Collagen I, RUNX-2, OSX, and Bone Sialoprotein (BSP) in hOB monolayers left untreated (CTL) or after treatment with deciduous tooth (DT) or permanent tooth (PT) T1 supernatants. Nuclei were counterstained with DAPI (blue) (original magnification 20×). Scale bar: 50 µm.
